# Ovarian adenocarcinoma metastasis mimicking psoas abscess on imaging: a case report

**DOI:** 10.11604/pamj.2020.36.231.21137

**Published:** 2020-07-29

**Authors:** Youssef Kharbach, Siham Rachidi Alaoui, Abdelhak Khallouk

**Affiliations:** 1 Urology Department, Mohammed VI Hospital, Faculty of Medicine, Abdelmalek Essaâdi University, 90000 Tangier, Morocco,; 2 Radiology Department, Mohammed VI Hospital, Faculty of Medicine, Abdelmalek Essaâdi University, 90000 Tangier, Morocco

**Keywords:** Malignant psoas syndrome, psoas abscess, metastasis, ovarian adenocarcinoma

## Abstract

Malignant psoas syndrome (MPS) is very rare with poor prognosis, and usually occurs in patients with advanced and recurrent cancer. Authors report herein the case of a 48-year-old female with history of neoadjuvant chemotherapy has been performed before hysterectomy with bilateral adnexectomy and ovariectomy for ovarian adenocarcinoma. She presented 18 months posttreatment with MPS due to a psoas abscess mimicking metastasis confirmed on computed tomography guided fine needle aspiration cytology.

## Introduction

Malignant psoas syndrome (MPS) is very rare with poor prognosis, and usually occurs in patients with advanced and recurrent cancer [1]. Its recognition is mandatory to allow the introduction of adequate therapies at early stages. Our case has the aim of presenting a rare case of malignant psoas syndrome revealing ovarian adenocarcinoma recurrence mimicking psoas abscess on imaging.

## Patient and observation

**Figure 1:**
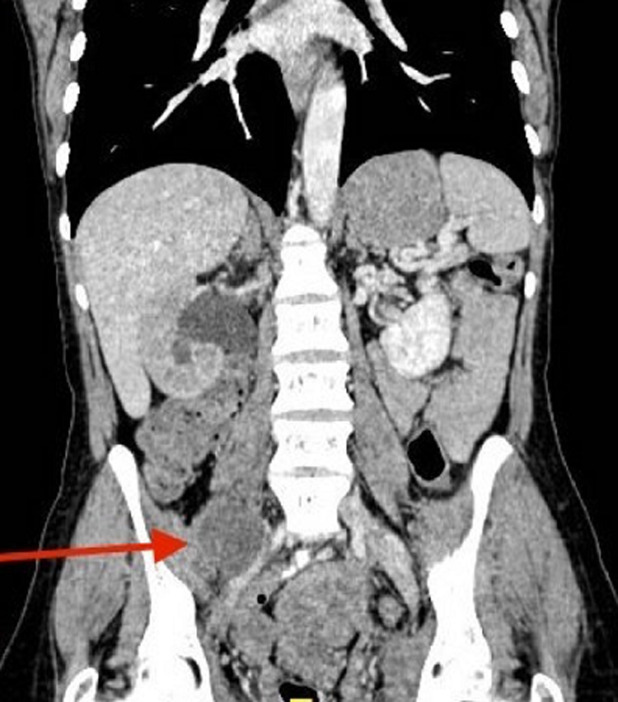
computed tomography (CT) image showing psoas abscess-like lesion with homolateral hydronephrosis

**Figure 2:**
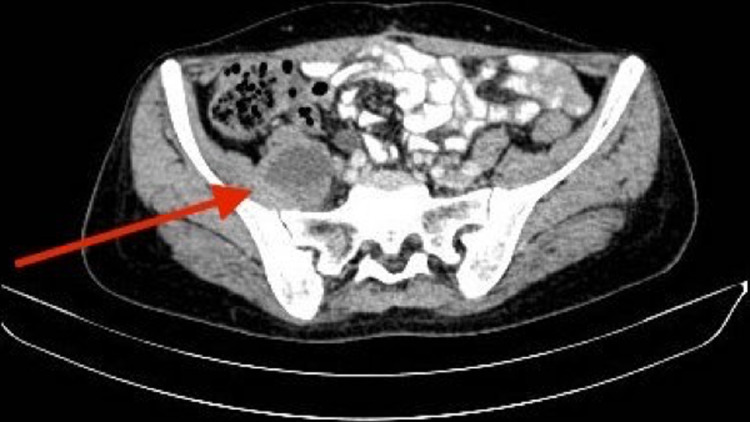
computed tomography showing a well-defined hypodense lesion in the right psoas muscle, measuring 4 x 3.6 x 3 cm

A previously healthy 48-year-old multiparous female had been diagnosed as a case of ovarian adenocarcinoma 18 months ago. A neoadjuvant chemotherapy has been performed before hysterectomy with bilateral adnexectomy and ovariectomy. At 18 months posttreatment the patient presented with complaints of low backache for the last 2 months. The pain was right-sided, extending from the lower back through the hip and thigh to inside the knee. Clinical examination identified painful flexion of the right hip and did not show any feature of local recurrence. The patient underwent computed tomography (CT) examination of the abdomen and the pelvis which showed multiple pelvic lesions, one of these lesions was measuring 5.5 cm and causing a medial dislocation of the right ureter with homolateral hydronephrosis. An associated well-defined hypodense lesion in the right psoas muscle, measuring 4 x 3.6 x 3 cm was also identified (Figure 1, Figure 2). Initially, a diagnosis of ovarian adenocarcinoma recurrence with right psoas abscess was made due to the patient history. Laboratory investigations revealed a haemoglobin level of 13.8 g/dl, total leukocyte count of 7,200 cells/mm^3^ with 61% neutrophils, 33% lymphocytes, 6% eosinophils, 0% monocytes and 0% basophils. Serum creatinine and blood urea levels were within normal limits. Subsequently, CT guided fine needle aspiration cytology of the right psoas lesion was performed, which revealed the presence of adenocarcinoma cells consistent with the diagnosis of metastatic adenocarcinoma.

## Discussion

MPS was first described by Stevens and Gonet in 1990, it´s a cancer-related syndrome that associates ipsilateral proximal lumbosacral plexopathy and painful hip flexion due to evident malignant involvement of the psoas [1]. It is a rare entity and its incidence is less than 1% [1], this is due to the several protective mechanisms against metastatic involvement that muscles have [2]. MPS usually occurs in patients with advanced and recurrent cancer [1], and female genital tract malignancies are the most frequent causes [1], it generally occurs as a result of systemic spread [3].

Metastasis in the psoas muscle might arise in the psoas lymph nodes located between the musculature and the spine [4]. Most lesions are incidentally discovered on follow-up helical CT and most of them are neither painful nor palpable [3,5], but the adoption of combined PET/CT should increase its incidence [5]. Psoas metastasis presents with a broad spectrum of radiological features [2,4,5], but abscess-like intramuscular lesion with central low attenuation and rim enhancement is the most frequent appearance of psoas metastasis [4]. Several reports have proposed approaches for the management of MPS, but no single, crucial protocol has been established [1]. Treatment depends on the clinical setting and the condition of the patient. Therapeutic options include radiotherapy, chemotherapy and surgical excision [1,3]. The survival of patients with MPS is very short with median survival duration of 5.5-10.7 months after diagnosis [1].

## Conclusion

Psoas metastases are rare. Very often, CT imaging could find abscess-like lesions especially in the absence of known primary cancer, this is why core needle biopsy is mandatory to confirm the diagnosis.
